# Association of bullous pemphigoid with acquired hemophilia A: a case report

**DOI:** 10.11604/pamj.2024.48.27.43722

**Published:** 2024-05-29

**Authors:** Ouadii Abakarim, Fatima Ezzahra Lahlimi, Illias Tazi

**Affiliations:** 1 Department of Clinical Hematology and Bone Marrow Transplantation, University Hospital Centre Mohammed VI, Faculty of Medicine and Pharmacy, Cadi Ayyad University, Marrakesh, Morocco

**Keywords:** Acquired hemophilia A, bullous pemphigoid, autoimmune diseases, case report

## Abstract

Acquired hemophilia A, a rare condition resulting in spontaneous bleeding without prior bleeding disorders, arises due to autoantibody-mediated inhibition of coagulation factor VIII and is typically associated with autoimmune, neoplastic, drug, or obstetric factors. We present the case of a 31-year-old woman with bullous pemphigoid, managed with corticosteroids since 2013, who presented spontaneous hemorrhagic manifestations. Upon admission, laboratory tests revealed hypochromic microcytic anemia, prolonged activated partial thromboplastin time, and a factor VIII level < 1%, indicative of acquired hemophilia A. Further assessments showed elevated Ristocetin cofactor activity, von Willebrand factor antigen, and a factor VIII inhibitor level of 665 BU. This underscores the importance of considering acquired hemophilia A in autoimmune dermatological conditions like bullous pemphigoid, highlighting the association between autoimmune disorders and coagulation abnormalities, particularly in cases of spontaneous hemorrhagic events.

## Introduction

Acquired hemophilia A is a serious condition that is still not well understood [1]. It is caused by an autoimmune condition in which there is sudden production of autoantibodies directed against the antihemophilic factor VIII (FVIII) in an individual without any family history of bleeding. The clinical presentation can range from a simple ecchymosis to a major bleeding syndrome. Unlike severe congenital hemophilia A, hemarthrosis is rare [2]. Acquired hemophilia is always surprising and sometimes fatal [3]. The association of acquired hemophilia A with autoimmune diseases is found in 20% of cases [4]. Bullous pemphigoid is an autoimmune blistering skin condition characterized by subepidermal blister formation on the skin and rarely on the mucous membrane. The association between acquired hemophilia A and bullous pemphigoid has been reported in approximately 2% of patients [5]. Here we report an observation of acquired hemophilia during pemphigoid, illustrating this rare association.

## Patient and observation

**Patient information:** a 31-year-old female patient, single, and had no personal or family history of benign or malignant hemopathy. The patient had been followed up in the dermatology department 9 years ago for bullous pemphigoid.

**Figure 1:**
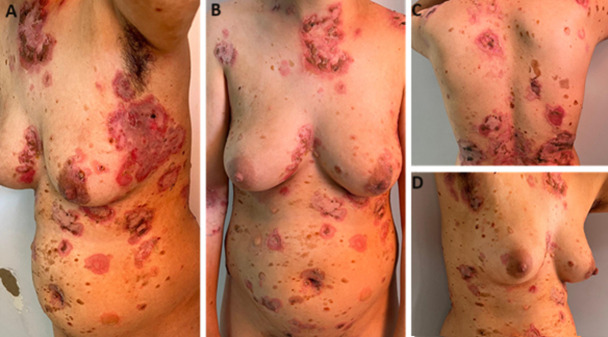
A, B, C, D) macroscopic appearance of bullous pemphigus with disseminated bullae ruptured on the trunk and in flexion zones with erythematous plaques

**Clinical findings:** disseminated bullae developing on the skin of the trunk and in the flexion zones and preceded by erythematous plaques (Figure 1). A positive diagnosis was confirmed by histological analysis and direct immunofluorescence of the skin lesions.

**Timeline of the current episode:** August 2013: the patient was administered prednisone-based corticosteroid therapy at a dose of 5 mg/day with good clinical improvement marked by the disappearance of bullous lesions. June 2022: the patient presented with a hemorrhagic syndrome with repeated epistaxis and ecchymosis, gingivorrhages and hemarthroses of both knees without anemic, infectious, or tumoral syndrome. July 2022: patient referral to clinical hematology department for further management.

**Diagnostic assessment:** a biological workup was performed, including a blood count that showed microcytic hypochromic anemia (hemoglobin: 9.7 g/dL, GMV: 77.9 fL and MCHF: 23.1 pg), normal platelet count, and normal leukocyte count. The hemostasis workup showed an activated partial thromboplastin time of 100 s (control: 34 s) without correction by the addition of control plasma and a normal prothrombin time of 95%. Further investigations revealed an antigenic WILLEBRAND factor at 344%, a WILLEBRAND factor activity at 182%, a factor VIII at 1% (normal between 60 and 150%) and an anti-factor VIII (anti-FVIII) antibody at 665 Bethesda units (BU)/ml (normal less than 0.6 BU/ml), confirming the diagnosis of acquired hemophilia A.

**Diagnosis:** the patient was diagnosed with acquired hemophilia A due to bullous pemphigoid.

**Therapeutic interventions:** therapeutic management was prescribed based on recombinant human factor VII at a dose of 90 μg/Kg every 4 h, but the patient could not obtain it because of the lack of financial means. An alternative treatment was initiated based on a general corticosteroid therapy (prednisone) at a dose of 2 mg/kg/day. Potassium and calcium supplements were prescribed during corticosteroid therapy.

**Follow-up and outcome of interventions:** hemorrhagic signs progressively regressed during corticosteroid treatment. The activated partial thromboplastin time, performed after 15 days of corticosteroid therapy, was 62s (control: 29s). A progressive decrease in corticosteroid therapy was initiated on the 21^st^ day of the initiation of treatment by reducing it by 5 mg/kg every 7 days until the therapy was ended. The evolution of our patient was favorable with the regression of the bleeding syndrome and a decrease in the activated partial thromboplastin time.

**Patient perspective:** during treatment, the patient was satisfied with the level of care provided to her.

**Informed consent:** the purpose of the study was explained to the patient, and informed consent was received before samples were collected. The patient was made aware that her medical records would be kept confidential.

## Discussion

Acquired hemophilia A, often revealed by mucocutaneous hemorrhages, is the most common acquired hemophilia [3]. This condition is defined by the appearance of a coagulation defect due to the presence of autoantibodies directed against factor VIII or antihemophilic A, resulting in a quantitative deficiency of this factor [6]. The positive diagnosis of acquired hemophilia A is based on the following criteria: the first criterion is the prolongation of the activated partial thromboplastin time, uncorrected by the addition of control plasma, according to the Rosner index. The second criterion is the decrease in Factor VIII (less than 30%, or 30 IU/dl). The third criterion was the presence of specific anti-factor VIII circulating anticoagulant autoantibodies titrated using the Bethesda- Nijmegen method. These autoantibodies are mainly polyclonal immunoglobulin G, which has a high affinity for FVIII [1]. They interact with the A2, A3, or C2 domains of FVIII and block its interactions with FIXa, phospholipids, and Willebrand factor, thus inducing a decrease in its coagulant activity [1]. The diagnosis ought to be considered in individuals experiencing sudden-onset bleeding, particularly those lacking any prior personal or family history of such incidents. Confirmation typically relies on specific assays detecting FVIII inhibitors [7].

Approximately 50% of acquired hemophilia A cases are idiopathic, whereas the other half are associated with various situations, including pregnancy, postpartum, autoimmune diseases, neoplasia, and drug reactions [8]. Acquired hemophilia A can be related to dermatological conditions such as psoriasis vulgaris, vitiligo, and squamous cell carcinoma [9]. However, the association of acquired hemophilia A with bullous pemphigoid is rare; to date, only 30 cases have been reported in the literature [9]. Bullous pemphigoid is an autoimmune dermatological condition characterized by autoantibodies directed against two skin proteins, BP180 and BP230, located in the dermal-epidermoid junction. The association of acquired hemophilia A with bullous pemphigoid is probably due to a cross-reaction of autoantibodies because of sequence homology between FVIII epitopes and the collagen XVII domain (BP180), a key component of the epidermal anchoring complex, which is also involved in the regulation of keratinocyte differentiation [4]. Bullous pemphigoid often precedes the onset of hemophilia (average delay of 6 months); the two conditions may also occur simultaneously [10]. In our observation, bullous pemphigoid and acquired hemophilia A appear to have evolved independently of each other, without a skin flare-up when the hemophilia was discovered.

The strategy of therapeutic management is based first on the treatment of bleeding complications according to the clinical points of call and the vital consequences; urgent management consists of the injection of activated human recombinant factor VII, activated prothrombin complexes, or human or porcine factor VIII. If the bleeding syndrome is moderate, antifibrinolytics are used as the first-line therapy [6,7]. Immunosuppressive therapy must be initiated with the aim of neutralizing antibodies; corticosteroid therapy is most often used alone or in combination with cyclophosphamide [10]. In our patient, the use of corticosteroid therapy alone was the only therapeutic option because it was impossible for the patient to obtain recombinant factor VII. The assessment of management is based on clinical (regression of the bleeding syndrome) and biological (blood count, activated partial thromboplastin time, factor VIII and anti-factor VIII autoantibodies) data [9].

## Conclusion

Our case presents a 31-year-old woman with bullous pemphigoid who developed acquired hemophilia A, underscoring the rare association between autoimmune dermatological conditions and coagulation abnormalities. The case highlights the importance of considering acquired hemophilia A in patients with autoimmune diseases presenting with spontaneous hemorrhagic manifestations, emphasizing the need for prompt diagnosis and tailored therapeutic interventions.
